# Osteoimmuno-brain axis: a bridge connecting osteoporosis and cognitive decline and its clinical significance in dementia and Alzheimer’s disease

**DOI:** 10.3389/fimmu.2026.1866071

**Published:** 2026-06-24

**Authors:** Guang Xu, Zheng Cheng, Yali Zhou, Liang Guo, Huihua Zhu, Zuojia Shen

**Affiliations:** Department of Orthopaedics, Jiangshan People’s Hospital, Jiangshan, Zhejiang, China

**Keywords:** Alzheimer’s disease, bone-derived factors, cognitive decline, dementia, inflammation, osteoimmuno-brain axis, osteoporosis

## Abstract

Osteoporosis and cognitive dysfunction, particularly Alzheimer’s disease (AD), frequently co-occur in the elderly population, suggesting shared pathophysiological links. The emerging concept of the “osteoimmune-brain axis” provides a framework that emphasizes the immune system as the central mediator connecting skeletal and cerebral pathology. This review explores how the osteoimmune system—the dynamic interface between bone and immune interactions—influences brain function through pathways including systemic inflammation, cytokine release, and bone-derived hormones. We systematically synthesize evidence from basic mechanisms to clinical studies, with a critical appraisal of the strength and directionality of the evidence. Key questions addressed include: whether the observed associations are causal or merely associative; which bone-derived factors have human validation; and whether the axis primarily operates as a unidirectional driver, a bidirectional amplifier, or an epiphenomenon of shared aging processes. This review aims to provide a balanced theoretical foundation for future research and for developing prevention strategies targeting both skeletal and neurodegenerative diseases.

## 1 Introduction

Osteoporosis and Alzheimer’s disease represent two of the most prevalent and debilitating age-related disorders, imposing a significant burden on global healthcare systems. Epidemiological studies have long suggested a clinical association between these conditions, with individuals diagnosed with one often exhibiting a higher risk of developing the other (1, 2). For instance, patients with Alzheimer’s disease (AD) have a high prevalence of osteoporosis (OP) and a substantial risk of osteoporotic fractures (3). Conversely, evidence indicates that patients with OP may have a higher risk of AD than those without (2). This bidirectional epidemiological link suggests the involvement of shared pathophysiological pathways rather than a simple causal relationship where one disease directly causes the other (1, 2). Traditionally, the skeleton was viewed merely as a structural scaffold, and the brain as the seat of higher cognitive functions. However, this paradigm has shifted dramatically. Bones are now recognized as dynamic endocrine organs capable of secreting factors that influence systemic physiology, including brain function (4). Concurrently, the brain is understood to exert regulatory control over peripheral organs, including bone. This redefinition has paved the way for the conceptualization of intricate inter-organ communication networks, such as the bone-brain axis, which describes a complex bidirectional signaling system connecting the skeletal and central nervous systems (5).

The foundation of this connection lies in the emerging field of osteoimmunology, which explores the interplay between the skeletal and immune systems. This interaction is central to the pathogenesis of both osteoporosis and neuroinflammation seen in Alzheimer’s disease. Bone is not an immunologically inert tissue; its marrow cavity is a primary site for hematopoiesis and immune cell development (6). Cells within the bone microenvironment, including osteoblasts, osteoclasts, osteocytes, and bone marrow-derived immune cells, secrete a plethora of signaling molecules. These bone-derived factors, or osteokines, along with immune-inflammatory mediators, can traverse the blood-brain barrier (BBB) or signal through alternative pathways to influence central nervous system (CNS) homeostasis (4, 7). Key osteokines implicated in this crosstalk include osteocalcin, sclerostin, and lipocalin-2. Osteocalcin, for example, has been shown in preclinical models to cross the BBB and regulate hippocampal development, neurotransmitter synthesis, and memory formation (4, 5). Sclerostin, a glycoprotein secreted by osteocytes that inhibits bone formation, is also expressed in the brain, where it may disrupt synaptic function and contribute to AD pathology by interfering with Wnt/β-catenin signaling—a pathway crucial for both bone formation and neuronal health (8). Lipocalin-2 is another bone- and immune-derived factor that can promote neuroinflammation. Furthermore, the immune system acts as a critical intermediary. Shared inflammatory pathways are a hallmark of both conditions. Chronic, low-grade systemic inflammation, or “inflammaging,” characterized by elevated pro-inflammatory cytokines like interleukin-6 (IL-6) and tumor necrosis factor-alpha (TNF-α), is a common feature of aging that can simultaneously drive bone resorption and neuroinflammation. Microglia, the resident immune cells of the brain, and bone marrow-derived macrophages exhibit similar dysfunctional activation states in AD and OP, respectively, perpetuating a cycle of tissue damage (9). This osteoimmune dialogue extends the concept to a “osteoimmuno-brain” axis, where immune cells originating from or regulated by the bone marrow microenvironment directly contribute to CNS pathology. For instance, skull bone marrow-derived myeloid cells can traffic to the meninges and brain parenchyma via recently discovered channels, directly influencing neuroinflammatory responses in conditions like sepsis-associated encephalopathy and potentially in chronic neurodegeneration (10, 11).

The molecular mechanisms facilitating this osteoimmuno-brain communication are multifaceted and involve hormonal, metabolic, and neural pathways. Hormonal dysregulation is a prominent shared feature. The decline of sex hormones, particularly estrogen during menopause, is a well-established risk factor for both postmenopausal osteoporosis and the increased incidence of AD in older women (12, 13). Follicle-stimulating hormone (FSH), whose levels rise sharply during menopause, has recently been implicated beyond reproduction. Preclinical and human data suggest that FSH has independent, direct effects on bone (promoting resorption), fat (increasing adiposity), and the brain, where it can accelerate amyloid-β and Tau deposition and impair cognition (14, 15). This positions FSH blockade as a potential therapeutic strategy targeting multiple age-related conditions simultaneously. Metabolic dysregulation forms another critical link. Conditions like type 2 diabetes mellitus (T2DM), obesity, and insulin resistance are common risk factors for both OP and AD (16, 17). Disrupted cellular glucose uptake, potentially mediated by reduced AKT kinase signaling, can lead to energy deficits in both osteoblasts and neurons, contributing to their dysfunction. Osteocalcin, in its undercarboxylated form, has been shown to regulate glucose metabolism and improve cognitive function, acting as a hormonal messenger from bone to brain. Neural pathways provide direct wiring for this axis. The autonomic nervous system, particularly the sympathetic nervous system (SNS), is innervated in bone tissue. Chronic stress or sympathetic overactivity can promote bone loss through β-adrenergic receptor signaling on osteoblasts, while also influencing systemic and neuroinflammation (5, 6). The vagus nerve, a component of the parasympathetic nervous system, is involved in the cholinergic anti-inflammatory reflex, which can modulate immune responses in both peripheral organs and the brain. Furthermore, shared intracellular signaling cascades, such as the Wnt/β-catenin and GSK-3β pathways, are crucially involved in bone anabolism and neuronal survival, and their dysregulation is a common thread in OP and AD pathology (8, 18). Lithium, a mood stabilizer, exemplifies a potential modulator of this axis by inhibiting GSK-3β, stabilizing β-catenin, and activating Wnt signaling, which may underlie observational findings of reduced dementia incidence and fracture risk in long-term users (18).

## 2 The concept and compositional basis of the osteoimmune-brain axis

### 2.1 Core elements and functions of the osteoimmune system

The osteoimmune system is fundamentally defined by the dynamic and reciprocal interactions between immune cells and bone cells within the skeletal microenvironment, a concept central to the interdisciplinary field of osteoimmunology. This crosstalk is vital for maintaining skeletal homeostasis and is critically involved in the pathogenesis of both skeletal and immune-mediated diseases (19). The core cellular interaction involves immune cells, such as macrophages, T cells, and B cells, with bone cells, including osteoclasts, osteoblasts, and osteocytes. A pivotal link is the monocyte/macrophage lineage, which serves as the precursor for osteoclasts, the bone-resorbing cells, thereby directly connecting immune cell origins to bone metabolism. The differentiation and activation of these osteoclasts are primarily governed by immune-derived cytokines, establishing a key immunologic mechanism in bone remodeling. The receptor activator of NF-κB ligand (RANKL) system is a quintessential example of this integration. RANKL, initially identified as a T cell-derived factor, is essential for osteoclastogenesis, and abnormalities in the RANKL/RANK/OPG (osteoprotegerin) axis lead to significant bone diseases, underscoring its foundational role in osteoimmunology (20). Beyond RANKL, a network of pro-inflammatory cytokines secreted by immune cells, such as tumor necrosis factor-alpha (TNF-α), interleukin-1 (IL-1), IL-6, and IL-17, directly regulates osteoclast differentiation and activity, driving excessive bone resorption and contributing to the pathogenesis of conditions like osteoporosis. These cytokines act as molecular links, with immune cells fine-tuning bone metabolism by mediating the balance between osteoclast and osteoblast function (21). Conversely, the skeletal system is not merely a passive target but an active immune organ. The bone marrow serves as the primary site for hematopoiesis, harboring hematopoietic stem cells (HSCs) and immune progenitor cells, and creating a specialized microenvironment essential for their maintenance and differentiation. This intricate multicellular network within the marrow means that dysregulation of immune-bone crosstalk in this environment can trigger systemic consequences, including exacerbated inflammation and even tumorigenesis. The systemic impact of skeletal immune status is further evidenced by studies showing that distinct systemic immune profiles, such as elevated levels of myeloid-derived suppressor cells (MDSCs) and interleukin-10 (IL-10), are correlated with and can predict impaired bone regeneration after trauma (22). This highlights that the bone marrow microenvironment’s state can systematically influence overall immune function and healing outcomes. Furthermore, the skeletal system’s role in immune system development is demonstrated by models where bone marrow transplantation into regenerating hematopoiesis enhances the reconstitution of the immune system, emphasizing the bone’s capacity to support immune cell engraftment and function (23). Thus, the osteoimmune system operates through a continuous feedback loop: immune cells regulate bone cell activity via cytokines, while the bone marrow microenvironment governs immune cell development and systemic immune responses, making it a critical axis for both skeletal integrity and immune homeostasis (24).

### 2.2 Communication pathways of the osteoimmune-brain axis: involvement of immune components

The communication between the skeletal and central nervous systems within the osteoimmune-brain axis involves multiple pathways in which immune components—such as immune cells, their secreted cytokines, and the bone marrow microenvironment—appear to play important roles. Unlike a conventional bone-brain axis that focuses primarily on endocrine or neural signals, the osteoimmune-brain axis emphasizes the potential contributions of immune-related mechanisms to this crosstalk. Based on current evidence, at least three types of pathways—humoral, neural, and cellular—have been proposed to mediate this communication, each of which may be influenced by osteoimmune factors.

Humoral pathway. Bone cells secrete various osteokines (e.g., osteocalcin, lipocalin-2, FGF23, sclerostin). Their production and release have been reported to be regulated by immune cells within the bone marrow. For instance, IL-1 and TNF-α derived from T cells and macrophages have been shown to modulate osteocalcin expression in preclinical studies, suggesting a possible link between skeletal immune activity and endocrine output. Pro-inflammatory cytokines (IL-6, TNF-α, IL-1β) produced during osteoimmune crosstalk can enter the circulation. Some of these mediators may cross the BBB or act on cerebrovascular endothelial cells, potentially contributing to secondary neuroinflammation (25). Thus, the humoral route may serve as a signaling system in which circulating mediators reflect, at least in part, the state of bone marrow immunity.

Neural pathway. Bone tissue receives innervation from sensory and sympathetic nerves. Immune cells within the bone marrow express receptors for various neurotransmitters, which could enable bidirectional interactions between the immune and nervous systems. Conversely, pro-inflammatory cytokines released during osteoimmune activation may activate local nerve endings. This sensory input might be relayed via afferent vagal or spinal pathways to brainstem and hypothalamic regions that are involved in regulating systemic inflammatory tone, for example through the cholinergic anti-inflammatory reflex (26). Therefore, the neural pathway could represent a route through which skeletal inflammation is linked to central autonomic control. The relevance of this pathway to the osteoimmune-brain axis lies in the observation that immune-derived mediators can trigger neural reflexes that, in turn, may influence peripheral immune responses.

Cellular pathway. A more direct route involves the migration of bone marrow-derived immune cells to the CNS. Anatomical studies have identified osseous channels, sometimes referred to as “skull channels,” that connect the bone marrow of the skull and vertebrae with the meninges. These channels have been shown to allow bone marrow-derived leukocytes (including monocytes, neutrophils, and other myeloid cells) to migrate directly into CNS compartments without passing through the peripheral circulation (27). Once in the brain, these cells have been observed to differentiate into microglia-like cells or to modulate resident microglial function, potentially influencing neuroimmune surveillance and neuroinflammation. Aging-related changes in the bone marrow, such as microvascular aging or altered hematopoiesis, may affect the phenotype of these migrating cells and could thereby be associated with chronic neuroinflammation (28). This cellular pathway is considered a distinguishing feature of the osteoimmune-brain axis compared with conventional bone-brain endocrine communication.

**Figure 1:**
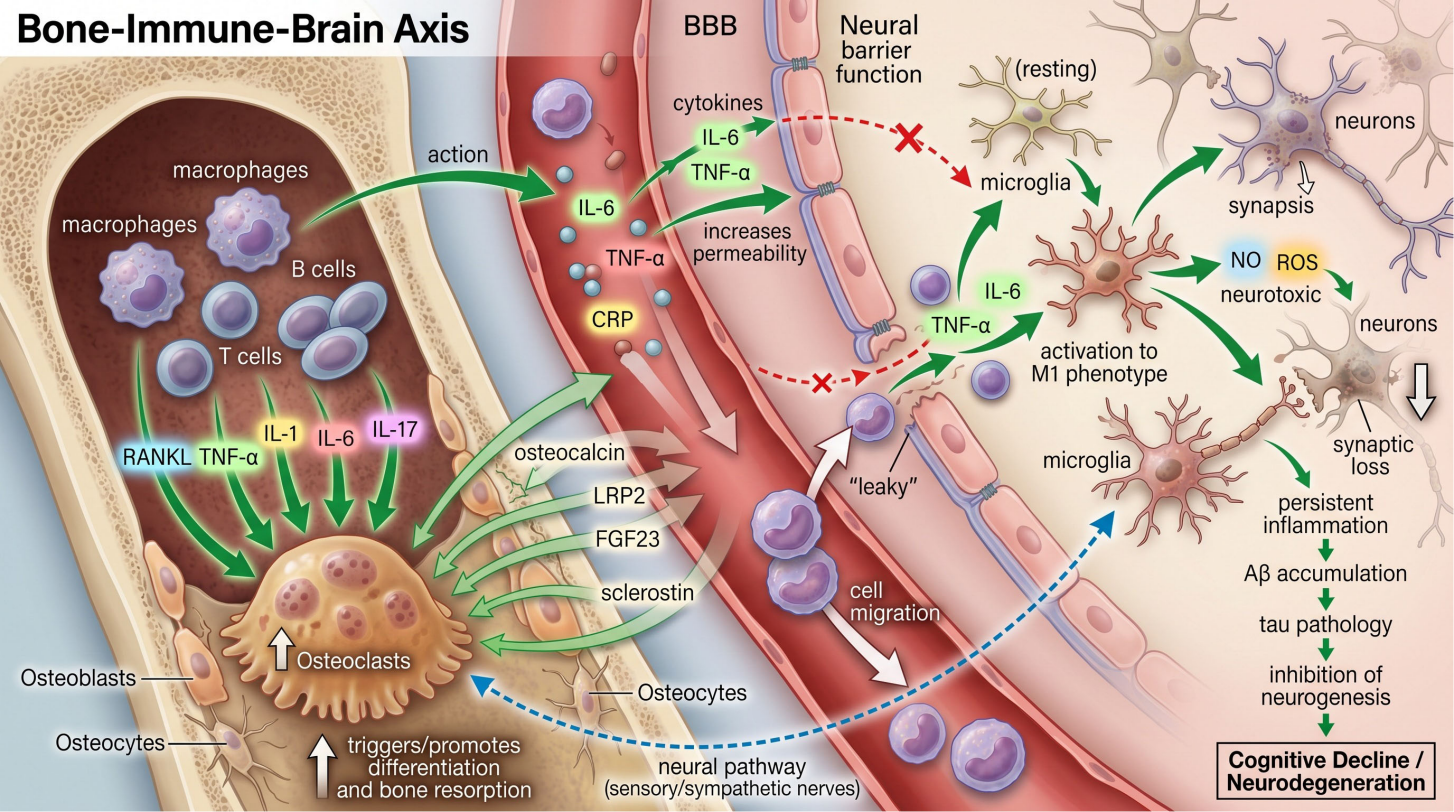
Osteoimmune-brain axis.

Together, these three pathways suggest that immune components may participate in bone-brain communication. The extent to which these pathways operate in humans and their direct contribution to cognitive health or disease remain to be determined. The overall framework of the osteoimmune-brain axis is schematically illustrated in **Figure 1**.

## 3 Systemic inflammation: the core linkage mechanism of the osteoimmune-brain axis

### 3.1 Chronic low-grade inflammation in osteoporotic conditions

Osteoporosis is increasingly recognized not merely as a metabolic bone disorder but as a condition deeply intertwined with a state of systemic, chronic, low-grade inflammation (SCLGI), often termed “inflammaging” (29). This inflammatory milieu is a hallmark of aging and is significantly exacerbated by key osteoporosis risk factors such as estrogen deficiency and advanced age (30). In postmenopausal women, the loss of estrogen initiates a cascade of immune dysregulation. Research demonstrates that ovariectomy in mice activates chronic low-grade inflammation mediated by long-lived dendritic cells and memory T-cells, which secrete pro-inflammatory cytokines like IL-17A and TNF-α, directly promoting bone catabolic activity and osteoporosis (31). This process is not exclusive to estrogen loss; aging itself dysregulates the immune system, leading to sterile low-grade inflammation where senescent, proinflammatory tissue-resident memory T-cells contribute to bone loss.

Bone-specific sources: The bone marrow cavity is a major reservoir of IL-6, TNF-α, and RANKL. Under osteoporotic conditions, activated T cells and macrophages in the marrow release these cytokines, which then enter the circulation. Importantly, bone-derived IL-6 may contribute disproportionately to systemic levels in conditions with high bone turnover, although this remains difficult to isolate from other sources. BBB disruption: Elevated systemic IL-6 and TNF-α, commonly observed in osteoporotic patients, increase BBB permeability by downregulating tight junction proteins (e.g., claudin-5, occludin), facilitating the entry of peripheral immune cells and neurotoxic substances into the brain parenchyma (32). Independence from age and general inflammation: A systematic review and meta-analysis confirmed that postmenopausal osteoporotic women have significantly higher circulating IL-6 and TNF-α compared to non-osteoporotic controls, independent of age (33).

### 3.2 Direct impact of inflammatory mediators on the blood-brain barrier and neurological function

The chronic low-grade inflammatory state characteristic of osteoporosis has profound implications for the central nervous system, primarily through the actions of circulating pro-inflammatory cytokines. Elevated systemic levels of mediators such as IL-6, TNF-α, and IL-1β, which are commonly observed in osteoporotic and inflammaging conditions, can compromise the integrity of the BBB (34). These cytokines increase BBB permeability, facilitating the entry of peripheral immune cells and neurotoxic substances into the brain parenchyma, thereby disrupting the delicate cerebral microenvironment (32). Once within the brain, these inflammatory factors directly activate the brain’s resident immune cells, the microglia. Under the influence of persistent inflammatory signals, microglia undergo a phenotypic shift from a resting, surveillance state to an activated, pro-inflammatory (M1) state (32). This activated microglia release a barrage of neurotoxic substances, including reactive oxygen species (ROS), nitric oxide, and additional pro-inflammatory cytokines (e.g., TNF-α, IL-1β, IL-6), which collectively inflict damage on neurons and synaptic structures, impairing cognitive function (35). This sustained neuroinflammation is recognized as a core driver in the pathogenesis of Alzheimer’s disease (AD). Inflammatory cytokines can accelerate the key pathological hallmarks of AD: they promote the deposition and aggregation of β-amyloid (Aβ) peptides and exacerbate tau protein hyperphosphorylation and tangle formation (32). Furthermore, chronic inflammation creates an environment hostile to neurogenesis, suppressing the birth of new neurons in the hippocampus, a brain region critical for learning and memory (35). The link between systemic inflammation from conditions like osteoporosis and brain pathology is reinforced by evidence showing that inflammatory markers such as IL-6 and TNF-α are not only elevated in osteoporosis but also serve as significant predictors and mediators in the progression to cognitive decline and dementia (33). This establishes a vicious cycle where bone-derived inflammatory mediators breach the BBB, ignite neuroinflammation, and drive neurodegenerative pathology, which in turn may further dysregulate systemic physiology. Therefore, the inflammatory mediators that are central to osteoporotic bone loss also act as direct effectors in the disruption of cerebral homeostasis, providing a mechanistic conduit through which skeletal disease can influence brain health and cognitive function.

## 4 Direct role of bone-derived hormones and factors in cognitive regulation

### 4.1 Central nervous system effects of osteocalcin

Osteocalcin (OCN), a non-collagenous protein secreted by osteoblasts, exists in carboxylated (cOC) and undercarboxylated (ucOC) forms, with the latter functioning as a bone-derived hormone capable of entering circulation (36). Importantly, the production of OCN can be modulated by inflammatory cytokines (e.g., IL-1, TNF-α) derived from bone marrow immune cells, suggesting that OCN operates within the osteoimmune regulatory network rather than as an autonomous bone-derived signal (37).

**Figure 2:**
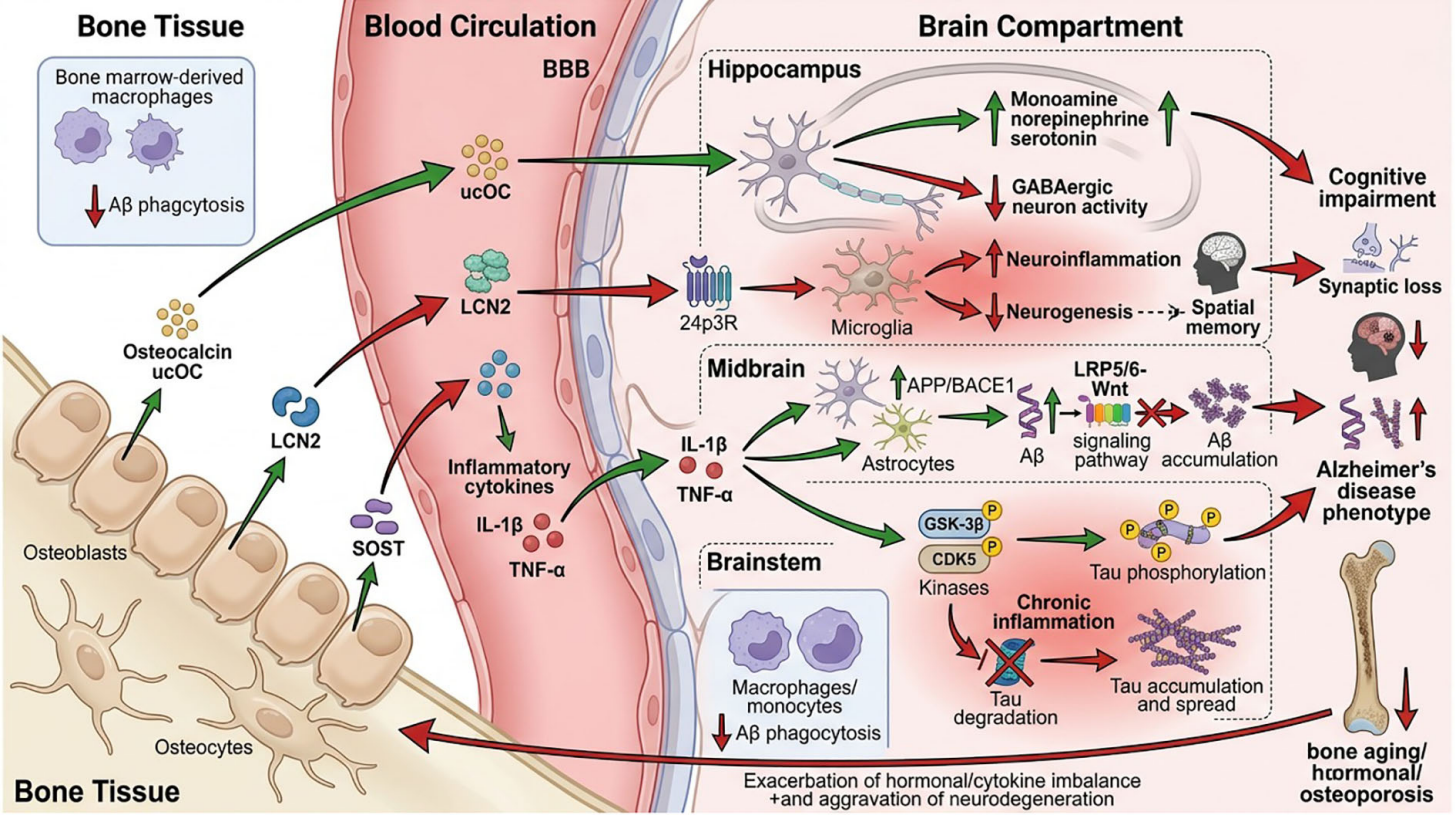
Role of bone derived hormones and factors in cognitive regulation and pathophysiology of Alzheimer’s disease.

A growing body of preclinical evidence has demonstrated that ucOC can cross the blood-brain barrier (BBB) and bind to specific receptors (e.g., GPR158, GPR37) in brain regions including the hippocampus, brainstem, and midbrain (38, 39). In animal models, ucOC has been associated with the regulation of monoamine neurotransmitter synthesis, inhibition of GABAergic activity, and improvements in learning and memory (40). Furthermore, OCN administration in transgenic mouse models of Alzheimer’s disease (AD) has been linked to reduced amyloid-β burden and ameliorated memory deficits (41, 42). The contributions of bone-derived hormones and factors to cognitive regulation and Alzheimer's disease pathology are summarized in **Figure 2**.

In human observational studies, higher plasma and cerebrospinal fluid OCN levels have been observed in patients with AD and mild cognitive impairment compared to cognitively normal individuals, and these levels have shown correlations with core AD biomarkers such as Aβ and tau (43). Similarly, lower serum OCN concentrations have been associated with brain microstructural changes and poorer cognitive performance in non-AD populations (44). Mendelian randomization studies have suggested a potential protective genetic association between higher OCN levels and reduced risk of AD (37, 45, 46). However, most human studies are cross-sectional, which limits causal inference.

### 4.2 The dual role of lipocalin-2

Lipocalin-2 (LCN2) is a glycoprotein secreted by activated osteoblasts and osteocytes, particularly in response to inflammatory stimuli (47). Of note, LCN2 production is highly sensitive to the local immune environment; inflammatory mediators such as IL-1β and TNF-α can upregulate LCN2 expression in bone cells, placing LCN2 at the intersection of osteoimmune and neuroinflammatory signaling (24). LCN2 can cross the BBB via the 24p3R receptor (48).

Once in the brain, LCN2 appears to exert context-dependent effects. In animal models, elevated LCN2 levels have been associated with hippocampal neuroinflammation, impaired neurogenesis, and spatial memory deficits (48, 49). This aligns with the concept that chronic low-grade inflammation—a hallmark of both osteoporosis and neurodegenerative conditions—may involve bone-derived signals such as LCN2 (50).

Human observational evidence for LCN2 is less consistent. Some studies have reported elevated CSF LCN2 levels in AD patients, with higher levels associated with lower cognitive scores and reduced cortical thickness (51). However, a systematic review and meta-analysis found no significant difference in CSF LCN2 between AD and control groups in the pooled analysis, and noted that peripheral blood LCN2 differences were influenced by specimen type (serum vs. plasma) and age (52). In contrast, CSF LCN2 was found to be elevated in vascular dementia compared to both controls and AD, suggesting a stronger link to vascular pathology. Post-mortem brain tissue studies have shown increased LCN2 expression in AD-related brain regions, predominantly in reactive astrocytes and microglia.

## 5 Specific involvement of the osteoimmune-brain axis in the pathophysiology of Alzheimer’s disease

### 5.1 Aβ metabolism and clearance

The potential link between the osteoimmune system and Aβ pathology has been explored through two main routes: inflammatory regulation of Aβ production and the involvement of bone marrow-derived immune cells in Aβ clearance. Pro-inflammatory cytokines elevated in osteoporotic conditions, such as IL-1β and TNF-α, have been shown in preclinical studies to upregulate amyloid precursor protein (APP) expression and promote BACE1 activity, a key enzyme in Aβ generation (53). These effects have been observed in neuronal and glial cell cultures and in animal models of neuroinflammation. However, direct evidence that bone-specific inflammation—as distinct from systemic inflammation of other origins—drives increased Aβ production in humans is lacking, as most studies have used general inflammatory stimuli rather than bone-targeted interventions. Regarding Aβ clearance, some preclinical studies suggest that peripheral monocytes or macrophages, whose function may be altered by the osteoporotic bone marrow microenvironment, exhibit reduced phagocytic capacity for Aβ (54). Currently, no direct human evidence demonstrates that bone marrow-derived immune cells significantly alter Aβ clearance in osteoporosis or AD. Thus, while it is biologically plausible that the osteoimmune axis influences Aβ metabolism, human validation remains lacking, and the direction of any such effect remains uncertain.

### 5.2 Impact on tau protein phosphorylation and neurodegeneration

The osteoimmune-brain axis may also influence tau pathology and neurodegeneration, primarily through inflammatory mediators and bone-derived factors that intersect with tau-related signaling pathways.

Inflammatory regulation of tau phosphorylation. Pro-inflammatory cytokines (IL-1β, TNF-α, IL-6) originating from the bone marrow microenvironment have been reported to activate kinases such as GSK-3β and CDK5 within the brain (55). These kinases are known to directly phosphorylate tau, promoting its aggregation into neurofibrillary tangles. Such effects have been observed in animal models of systemic inflammation and in cell culture studies. However, most of these models do not specifically isolate bone-derived inflammation from other sources (e.g., adipose tissue, gut). Bone-derived factors and tau. Among bone-derived factors, sclerostin (SOST) has been investigated for its potential to interfere with Wnt/β-catenin signaling, a pathway crucial for both bone formation and neuronal health (56). Disruption of Wnt signaling has been associated with increased tau phosphorylation and synaptic dysfunction in preclinical models. However, direct evidence that bone-derived SOST crosses the blood-brain barrier and promotes tau pathology in humans is absent.

In summary, preclinical studies suggest plausible mechanisms by which osteoimmune signals could contribute to tau pathology and neurodegeneration, but direct human evidence is sparse. The field awaits well-controlled longitudinal studies and, ideally, bone-specific interventions to clarify whether the osteoimmune-brain axis plays a causal role in tau-related neurodegeneration.

## 6 Clinical epidemiology and translational medicine evidence

### 6.1 Epidemiological association between osteoporosis and cognitive impairment/dementia risk

Multiple meta-analyses have confirmed a bidirectional association between osteoporosis and cognitive decline. A systematic review and meta-analysis of eight studies (136,222 participants) found that patients with osteoporosis had a significantly increased risk of cognitive impairment (57). Another meta-analysis of ten studies concluded that patients with cognitive impairment, particularly those with AD, have a 1.7-fold increased risk of osteoporosis compared to controls (58). A more recent meta-analysis of fifteen studies confirmed this bidirectional link (59). The association is especially pronounced in women. A community-based study reported that spinal and total hip osteoporosis were associated with a 1.83-fold and 2.24-fold increase, respectively, in the risk of cognitive impairment among women, a relationship not observed in men (60). Longitudinal data from the English Longitudinal Study of Ageing (ELSA) and the Health and Retirement Study (HRS) show that osteoporosis is associated with lower baseline memory scores and a steeper annual decline in memory, particularly in women with hypertension (61). Nationwide cohort studies in older women have consistently demonstrated that osteoporosis is associated with increased risks for all-cause dementia, AD dementia, and vascular dementia (62, 63). For example, a study of 131,872 Korean women aged 66 found that osteoporosis was associated with a 14% increased risk of all-cause dementia and a 42% increased risk of vascular dementia over a decade. Similarly, a Japanese study found that older women with low BMD had a 58% higher risk of all-cause dementia and a 61% higher risk of AD over approximately 30 months (64). In patients with type 2 diabetes mellitus, osteoporosis is also correlated with higher prevalence and severity of cognitive impairment.

Acute skeletal events, particularly hip fractures, are associated with accelerated cognitive decline and increased dementia risk. A meta-analysis found that a history of fractures was associated with a 28% increased risk of subsequent dementia (65). Hip fracture has been identified as a predictive marker for dementia risk (66). The presence of dementia at the time of hip fracture compounds adverse outcomes, including higher morbidity, mortality, and postoperative delirium (67–69). Longitudinal studies further indicate that the rate of bone loss is temporally correlated with the speed of cognitive decline, supporting shared systemic aging pathways rather than a simple unidirectional cause-and-effect relationship (70, 71).

### 6.2 Imaging correlations between bone and brain

Imaging studies provide structural evidence for the bone-brain connection by demonstrating correlations between skeletal integrity and brain morphology. A voxel-based morphometry study found that lower femoral neck BMD was correlated with reduced gray matter volume in the left precuneus, a brain region vulnerable to AD pathology, independent of age, sex, and global cognitive scores (72). Another multimodal MRI study in older women with suspected mild cognitive impairment found that lower BMD was associated with reduced subcortical cerebral blood flow and lower total brain parenchymal volume; additionally, reduced vertebral bone marrow perfusion and increased marrow fat content were linked to reduced brain volume (73). Total body BMD has also been significantly correlated with cognitive function in older adults, especially among women and those aged 60–80 years (74). Interestingly, this association may have early-life origins, as a study in children aged 6–7 years found that occipitofrontal circumference (a proxy for brain volume) was positively associated with whole-body BMD, suggesting common early-life determinants for skeletal and neural development (75). Collectively, these imaging findings reinforce the concept of a physiological link between bone and brain health, where structural indices of one system may inform about the status of the other.

## 7 Therapeutic implications and future directions

### 7.1 Current evidence for potential neuroprotective effects of anti-osteoporosis therapies

The hypothesis that anti-osteoporosis medications may confer neuroprotective effects has gained attention, but current evidence remains largely observational or preclinical, and no causal conclusions can be drawn.

For bisphosphonates, a large population-based study in Hong Kong reported an association between nitrogen-containing bisphosphonate use and a lower risk of Alzheimer’s disease and related dementias (76). This finding, however, is observational and subject to residual confounding such as healthier user bias. The proposed mechanism—reduction of systemic inflammation via suppression of bone resorption—is plausible but remains unproven in humans (77). No randomized controlled trial has confirmed a cognitive benefit, and direct evidence for effects on Aβ or tau pathology is absent. The RANKL inhibitor denosumab effectively prevents fractures, its modulation of the RANKL/RANK pathway, which is also involved in neuroinflammation, warrants future investigation., but no clinical recommendation for cognitive protection can be made at present (78).

Preclinical studies have shown that teriparatide enhances neurological recovery, reduces oxidative stress, and protects blood-brain barrier integrity in rat models of spinal cord injury and ischemic stroke (79–81). These findings suggest a potential dual action on bone and neural tissue, but human data on cognitive outcomes in osteoporotic patients are lacking. Nutritional supplements commonly recommended for bone health, such as vitamin D, calcium, and various phytochemicals (e.g., phytoestrogens, atractylenolides, chrysoeriol, prenylflavonoids), have been associated with neuroprotective effects in some pharmacological studies (82–85). However, the evidence linking these agents directly to the osteoimmune-brain axis is indirect and derived primarily from *in vitro* or small animal experiments. None of these supplements have been demonstrated in clinical trials to confer cognitive protection specifically through bone-brain mechanisms.

In summary, current evidence supports hypothesis-generating observations rather than established clinical applications. Clinicians should not prescribe anti-osteoporosis drugs solely for cognitive protection based on existing evidence.

### 7.2 Future therapeutic strategies targeting the osteoimmune-brain axis

One direction is the systematic evaluation ofdual-effect molecules. Natural compounds such as icariin, icaritin, epimedin B, luteolin, mogrol, and verbascoside have demonstrated both anti-osteoporotic and neuroprotective properties in preclinical studies (86–91). These findings support further testing in animal models that combine osteoporosis and cognitive impairment (e.g., ovariectomized APP/PS1 mice), with assessment of blood-brain barrier penetration and dose-response for both skeletal and cognitive endpoints. A complementary approach is the development of bone-targeted drug delivery systems. Bone-targeted nanomedicines (e.g., Nano-MP) and liposomal formulations have been shown to deliver therapeutic agents selectively to bone or injured neural tissue, thereby reducing systemic side effects such as muscle atrophy**Figure 1** and osteoporosis (92, 93). Future studies should test these delivery systems in animal models of combined osteoporosis and neurodegeneration.

Another emerging area is the investigation of bone-derived exosomes as natural signaling vehicles. Bone cells release exosomes carrying miRNAs, proteins, and other bioactive molecules that can mediate remote organ communication. Nutritional supplements and neuroprotective diets and their potential clinical significance in post-stroke rehabilitation (52).

Preclinical evidence suggests that such exosomes may influence brain pathology, although specific findings vary across studies (100). Future research should isolate and characterize exosomes from osteoporotic versus healthy bone, and test their effects on microglial function, synaptic integrity, and cognitive behavior in relevant animal models.

At the clinical level, integrated management strategies are needed. Given the documented disparities in osteoporosis diagnosis and treatment among individuals with cognitive impairment, combined bone density screening and cognitive assessment should be implemented in geriatric populations (94). Multidimensional geriatric assessments, such as the Multidimensional Prognostic Index, can help personalize anti-fracture therapy decisions (95). For patients with advanced dementia, deprescribing conversations regarding bisphosphonates should prioritize quality of life over life expectancy (96). Finally, lifestyle interventions remain the most accessible and low-risk strategy. Progressive resistance and weight-bearing exercise have separately been shown to benefit bone and cognitive health (97, 98). Nutritional strategies, including anti-inflammatory and neuroprotective diets, also support brain health (99). Future trials should examine the combined effect of exercise and dietary interventions on both skeletal and cognitive outcomes in older adults with low bone density and subjective cognitive decline.

## 8 Conclusion

The concept of the osteoimmune-brain axis represents a paradigm shift in understanding the interplay between the skeletal and central nervous systems, particularly in the context of aging-related pathologies. As an emerging interdisciplinary framework, it provides a plausible explanation for the shared biological underpinnings of osteoporosis and cognitive decline, including Alzheimer’s disease (AD). Nevertheless, a balanced appraisal of the current evidence is necessary before this framework can be considered clinically translatable.

The core premise that bone functions not merely as a structural organ but as a dynamic endocrine and immune regulator is well supported by preclinical studies. The axis operates through three interconnected conduits: systemic inflammation, bone-derived hormones and factors, and neural pathways. Osteoporosis is increasingly understood as a state of chronic, low-grade osteoimmune activation, characterized by elevated pro-inflammatory cytokines (e.g., IL-1β, IL-6, TNF-α) and altered osteokines such as osteocalcin and lipocalin-2. Preclinical evidence suggests that these systemic changes can compromise the blood-brain barrier, promote neuroinflammation, and exacerbate AD-related pathologies, including Aβ aggregation and tau hyperphosphorylation. This mechanistic cascade offers a coherent explanation for the epidemiological association between low bone mineral density and dementia.

Despite the strength of preclinical data, human evidence remains predominantly correlational. Most clinical studies are cross-sectional and cannot establish causality. Furthermore, the directionality of the observed association remains ambiguous. Cognitive decline itself may lead to reduced physical activity, poorer nutritional intake, and increased fall risk, all of which can lower bone mineral density. Thus, the bone-brain association may reflect a bidirectional pathological amplifier rather than a unidirectional causal pathway from osteoporosis to dementia. Shared confounders—including aging, frailty, systemic inflammaging, and common genetic risk factors such as APOE4—cannot be excluded. The role of specific osteokines in humans remains unsettled; for instance, the neuroprotective effects of undercarboxylated osteocalcin reported in mice await confirmation in human studies, and the context-dependent actions of lipocalin-2 (both detrimental and protective in different settings) require further delineation. Claims that existing anti-osteoporosis drugs (e.g., bisphosphonates, denosumab) exert direct neuroprotective effects remain speculative, as no randomized controlled trials with cognitive endpoints have been completed to date.

From a clinical perspective, the osteoimmune-brain axis re-contextualizes existing anti-osteoporotic therapies, encouraging the analysis of cognitive outcomes in osteoporosis trial databases and the design of prospective studies that simultaneously assess bone and brain health. More ambitiously, the framework suggests novel therapeutic directions, such as modulating osteokine pathways (e.g., enhancing osteocalcin signaling) or developing bone-targeted drug delivery systems for neuroprotective agents. However, these strategies are currently at the hypothesis stage and require rigorous preclinical and clinical validation.

Looking forward, the field must transition from association and mechanism to intervention and translation. Priority research directions include: (1) conducting rigorous prospective cohort studies with deep phenotyping (including imaging and fluid biomarkers for both bone turnover and neurodegeneration) to map the temporal dynamics of the axis in humans while adjusting for potential confounders; (2) initiating well-designed randomized controlled trials to test whether improving bone health—through pharmacological, exercise, or nutritional interventions—can slow cognitive decline or reduce dementia incidence; (3) employing advanced omics technologies (genomics, proteomics, metabolomics) to identify shared molecular signatures and causal pathways, including through Mendelian randomization approaches; and (4) fostering interdisciplinary collaboration among rheumatologists, endocrinologists, neurologists, and geriatricians to develop integrated models of patient care.

In summary, the osteoimmune-brain axis provides a powerful, mechanistic framework that moves beyond coincidence to explain the comorbidity of osteoporosis and cognitive impairment. It offers a more holistic understanding of aging by integrating systemic inflammation with bone-derived signals. However, important questions regarding human causality, directionality, and therapeutic efficacy remain unresolved. The ultimate impact of this framework will depend on its ability to generate testable hypotheses and, ultimately, to inform novel strategies for preserving both musculoskeletal and cognitive health in the aging population.
